# Differentiating Rhinitis in the Paediatric Population by Giving Focus on Medical History and Clinical Examination

**DOI:** 10.3390/medsci7030038

**Published:** 2019-02-26

**Authors:** Maria Doulaptsi, Noriaki Aoi, Hideyuki Kawauchi, Athanasia Milioni, Alexander Karatzanis, Emmanuel Prokopakis

**Affiliations:** 1 Department of Otorhinolaryngology-Head and Neck Surgery, University of Crete School of Medicine, GR-71003 Crete, Greece; mdoulaptsi@gmail.com (M.D.); athanassia.milioni@gmail.com (A.M.); akaratzanis@yahoo.com (A.K.); 2 Department of Otorhinolaryngology-Head and Neck Surgery, University of Shimane School of Medicine, Matsue 690-8504, Japan; nori-aoi@med.shimane-u.ac.jp (N.A.); kawauchi@med.shimane-u.ac.jp (H.K.)

**Keywords:** allergic rhinitis, infectious rhinitis, non-allergic rhinitis, rhinosinusitis, medical history, diagnosis, anterior rhinoscopy, nasal endoscopy

## Abstract

Chronic rhinitis is defined as an inflammation of the nasal epithelium, and is characterized by the presence of two or more specific nasal symptoms including obstruction, rhinorrhea, sneezing, and/or itching for at least 12 weeks. In childhood, this clinical entity is very common and carries a significant socioeconomic burden. The impact on the physical, social, and psychological well-being of family cannot be underestimated. Rhinitis is an umbrella term which includes different phenotypes of rhinitis with distinct underlying pathophysiologic mechanisms. In most cases the diagnosis of rhinitis is rather straightforward; however, sometimes when based on clinical symptomatology, characterization may be challenging. Herein, we provide guidance for getting all the data needed for the differential diagnosis of rhinitis based on medical history and clinical examination.

## 1. Introduction

Rhinitis is defined as an inflammation of the nasal epithelium causing two or more specific nasal symptoms such as rhinorrhea, obstruction, sneezing, and/or itching [1]. Based on symptoms’ duration, it can be classified as acute or chronic. Chronic rhinitis is characterized by the presence of two or more symptoms for at least 12 weeks. Rhinitis is an umbrella term; it can be distinguished in different categories, based on clinical characteristics and underlying pathology [2]. Current data support the classification into the following types: infectious rhinitis, allergic rhinitis (AR), and non-allergic rhinitis (NAR) [3,4,5,6]. Accordingly, some patients may have the so-called mixed rhinitis with features which overlap [3]. Chronic rhinitis appears to be very common in the paediatric population, reaching up to 45% in some regions [4]. In Western countries, hygiene hypothesis could explain in part the increased burden of allergic rhinitis [5]. It poses a significant socioeconomic burden, affecting both patient and family’s quality of life and well-being [6]. 

In the paediatric population, limitations in history taking, clinical examination, and laboratory/imaging investigation make the diagnosis of rhinitis a challenging process. For instance, we rely on parental accuracy for the information provided as medical history, especially with preschool-aged patients. Physicians refrain from submitting young patients to multiple tests (i.e., endoscopy, allergy tests, computed tomography (CT)) in order to diagnose, characterize, and differentiate rhinitis from other diseases. The lack of cooperation is another major issue, especially for younger patients. Additionally, co-morbidities related to rhinitis, should be sought and identified to lead to correct diagnosis and management. Conjunctivitis, chronic rhinosinusitis (CRS), asthma, adenoidal hypertrophy, sleep, learning, and speech disorders, as well as otitis media with effusion appear to be the most common co-morbidities of rhinitis in paediatric population. Physicians should also be aware of other rare clinical entities mimicking chronic rhinitis in paediatric patients, like immunodeficiency, cystic fibrosis (CF), and primary ciliary dyskinesia (PCD) [7,8]. Herein, we assess key features of the diagnostic process of rhinitis in the paediatric population. Special focus was given to medical history, inspection, and physical examination. Diagnostic tests and treatment are beyond the scope of this study.

## 2. Medical History and Clinical Examination

In general, the diagnosis of rhinitis in clinical practice can be made based on a detailed medical history as well as a simple physical examination accompanied with laboratory investigation for detection or exclusion of a clinically relevant sensitization to airborne allergens [1]. In the differential diagnosis, a detailed clinical history is of great importance to obtain all the information required to make the diagnosis of rhinitis, to characterize the type, to exclude other pathologies and co-morbidities, to deliver appropriate treatment, and to establish the best long-term management for the patient [9]. In Table 1, the most common causes of chronic rhinitis in the paediatric population are presented. Recognizing possible rhinitis co-morbidities is essential, as many of those may have escaped parental attention. After a series of questions, an experienced clinician should be able to assume a possible diagnosis and guide clinical examination and laboratory and/or imaging investigation to confirm the speculated pathology. Table 2 includes basic questions which need to be answered during history taking. Inspection, of both nose and face is of critical importance, especially in paediatric patients, and can give us valuable information even at first site [9]. For instance, a child with the characteristic long face syndrome should raise the suspicion of chronic nasal obstruction and mouth breathing, together with underlying causes. Table 3 contains typical signs and symptoms observed during clinical examination and their possible underlying aetiologies. 

Clinical evaluation should include a thorough examination of ear, nose, oral cavity, and pharynx, as well as neck palpation, and chest auscultation [10]. In infants, a gross appreciation of the anterior part of the nose can be done by pushing up the tip of the nose. This move will reveal the septum and the head of the inferior turbinates. Anterior rhinoscopy will allow the inspection of the anterior parts of the nose. It can be easily performed by all physicians with the use of a nose speculum and a forehead light or with a simple otoscope. Anterior rhinoscopy may reveal nasal secretions (transparent or discoloured), crusting, mucosal oedema, anatomic asymmetries, foreign bodies, presence of large polyps, or perforation of the anterior part of the septum. Nasal airflow during expiration can be tested by using a cold mirror or a metallic tongue depressor. Asymmetrical or total lack of fogging indicates unilateral or bilateral nasal obstruction, respectively [9]. 

Every time a meticulous assessment of the entire nasal cavity is warranted, nasal endoscopy should be carried out. It allows better visualization of both anterior and posterior parts of the nose, as well as septum and nasopharynx. It can be performed by either rigid or flexible endoscopes; though rigid scopes are considered superior in nasal cavity assessment and more patient friendly. Local administration of anaesthetic and decongestant is usually needed to facilitate the procedure. Young age is not considered a contraindication to nasal endoscopy, and it can be readily performed after the age of 2. Use of special flexible endoscopes (2.7 mm diameter) is better tolerated in smaller children. Nasal endoscopy allows assessment of nasal anatomy and variations, the state of nasal mucosa (inflamed or allergic), the patency of nasal ostiums, the presence and type of secretions (transparent, discoloured), the presence of small polyps, hypertrophic adenoids, foreign bodies, or nasal tumours [1,9,11]. 

## 3. Infectious Rhinitis

Viral infections are the most common cause of acute infectious rhinitis and these episodes can last up to 7–10 days and are usually self-limited. Human rhinovirus (more than 100 serotypes) is the most common cause, accounting up to 50% viral rhinitis episodes in children and adults. Respiratory syncytial virus (RSV), influenza virus, parainfluenza virus, adenovirus, enterovirus, and coronavirus have also been implicated in the pathogenesis of acute infectious rhinitis [11,12,13]. It is estimated that an infant can typically have up to 11 upper respiratory tract infection episodes per year. The number usually diminishes with age, with generally around eight episodes at preschool age and four at school age [10]. Bacterial contamination can prolong the course of acute viral rhinitis. Studies have reported that up to 10% of viral rhinitis cases can be complicated by superadded bacterial infections [14]. *Streptococcus pneumoniae*, *Moraxella catarrhalis*, and *Haemophilus influenzae* are the most frequent micro-organisms in bacterial rhinosinusitis. Polymicrobial flora is present in about one-third of cases, while anaerobes are often present in odontogenic infections in older children [14]. 

Chronic infectious rhinitis is usually associated with chronic sinusitis or adenoiditis. Bacteriology in children includes alpha-haemolytic streptococci, *S. pneumoniae*, *H. influenzae*, *M. catarrhalis*, *Staphylococcus aureus*, as well as anaerobes (*Peptostreptococcus, Bacteroides, Prevotella*, and *Fusobacterium* species). Fungal infections are rare in the paediatric population [15]. The presence of bacterial biofilms in adenoidal tissue or in sinonasal mucosa is suggested to be linked with chronic infections and inflammations of the nose and paranasal sinuses in adults. Biofilms are thought to contribute to the pathophysiology of chronic sinonasal infections in children as well, but more studies are needed to clarify this [16].

Cardinal symptoms of infectious rhinitis include mucopurulent discoloured secretions accompanied by nasal congestion, while sneezing, pruritus, and itching are usually absent. History of nose picking may be suggestive of rhinitis of infectious origin. Clinical examination with anterior rhinoscopy (±endoscopy) reveals a red mucosa, accompanied with mucopurulent secretions and/or crust formation. Nasal septal perforation or a foreign body may also be observed, which could be the underlying cause of rhinitis in these cases. The presence of infectious rhinitis does not rule out the co-existence of other types of rhinitis [11]. Allergen sensitization with or without clinical allergy was linked with prolonged inflammatory changes after virus-induced upper respiratory tract infection in the adult population [17]. 

Chronic mucopurulent secretion should also raise suspicion for adenoidal hypertrophy, chronic rhinosinusitis, anatomical abnormalities, primary ciliary dyskinesia, cystic fibrosis, and other clinical entities [10]. 

## 4. Allergic Rhinitis

In allergic rhinitis, nasal symptoms are classically associated with exposure to an allergen to which the patient is sensitized [1]. Classic symptoms are nasal obstruction, watery rhinorrhoea (anterior or posterior), pruritus, and sneezing (especially paroxysmal) which typically occur immediately after allergen exposure and may last for hours. By definition, the presence of two or more of the aforementioned symptoms for >1 h on most days indicates AR [1,18]. Red, itchy eyes are suggestive of conjunctivitis of allergic origin, which strongly correlates with AR. In young children, AR may have atypical presentation [1]. Reduced hearing, pruritic mouth and throat, sleep disorders, cough, poorly controlled asthma, and eczema are potential symptoms indicating AR [10]. History again is of great importance. Symptoms which occur on only one side of the nose, thick discoloured secretions, nasal obstruction without other symptoms, facial pain, epistaxis, and loss of smell are typically not found in AR. The presence of any one of them suggests that an alternative diagnosis is possible and should be investigated. Response to previous treatment should also be taken into account, as this may provide additional clinical insight [10]. 

“Atopic march” represents a sequence of atopic disorders from atopic dermatitis in infants to allergic rhinitis and asthma in childhood [19]. Manifestations of the disease and comorbidities should always be sought during history and physical examination. Bear in mind, those comorbidities may be undiagnosed and have escaped parental attention. Symptoms and signs suggestive of conjunctivitis, asthma, eczema, rhinosinusitis, pollen food allergy, hearing, and sleep disorders should be sought. History of coughing, shortness of breath, and wheezing should alert us for asthma co-existence. Always assess speech and language development delay, high TV volume, poor concentration, and performance at school. Assess symptoms and signs indicative of disturbed sleep such as apnoea, snoring, and day tiredness [10]. The nose is located strategically in the airway and is functionally linked to paranasal sinuses, eyes, middle ears, pharynx, larynx, and lower airway. Hence, apart from classic symptoms of AR, presenting features may be conjunctivitis, chronic cough, mouth breathing, nasal speech, snoring, pruritic mouth and throat, and/or pain of ears on pressure changes. 

Inspection can give us additional clue to further support AR diagnosis. Typically, we can see a child rubbing and wiping his or her nose in an upwards or transverse manner with the fingers, palm, or back of the hand (*allergic salute*). As a result, we may observe the characteristic horizontal line near the tip of the nose (*allergic crease*) [20]. Dark discoloration of the periorbital skin (*allergic shiners*) [21], folds of the lower eyelids (*Dennie–Morgan lines*) may further support our diagnosis [10]. Signs of conjunctivitis such as red, watery eyes, hyperaemia of conjunctiva, and eye rubbing are typically present; especially in pollen-induced AR. Remember that allergic conjunctivitis is the most frequent comorbidity of AR, especially in children. Up to almost three-quarters of AR patients will have allergic conjunctivitis, and, among patients with allergic conjunctivitis, the prevalence of AR may reach 97% [22].

Clinical examination with anterior rhinoscopy will help us to build up and strengthen our diagnosis, and to avoid unnecessary referrals, testing, or treatment. Enlarged turbinates with pale, purplish, and bluish mucosa as well as thin watery or mucoid secretions in the nose are indicative of rhinitis of allergic origin [23]. A thoroughly performed ear examination may reveal tympanic membrane retraction or features of chronic otitis media with effusion. Concerning the possible association of AR and otitis media with effusion, current evidence remains unclear. However, a transient eustachian tube obstruction was demonstrated after nasal challenge with aeroallergens [24,25,26]. The pharynx may reveal a cobblestone appearance of the posterior pharyngeal wall, and hyperplasia of tonsils and/or adenoids. Regarding adenoid hypertrophy in AR patients, evidence is inconsistent. Children with AR have repeatedly shown increased prevalence of adenoid hypertrophy, however, children with upper airway obstruction do not consistently show an increased sensitivity in airborne allergens [27,28]. The significance of different age peaks for several pathologies should not be underestimated, and thus should be considered. Particularly, it is known that the prevalence of AR in the paediatric population increases with age, with its peak occurring in adolescence [29,30]. Contrariwise, infectious rhinitis prevalence is remarkable high in early life, while it steadily decreases over time. For adenoid hypertrophy, peak age is considered to be the 4–7 years age group [11].

Children with AR, as well as chronic mouth breathers, may present with characteristic facial feature changes, such as narrowing of the hard palate, gothic arch, discoloration of frontal incisors, dental malocclusion (crooked teeth), everted upper lip, and loss of nasolabial fold, which all lead to a narrow, long face [10,18,22]. However, we should be careful interpreting these changes, as they may appear in every condition with chronic nasal obstruction and mouth breathing, like adenoid hypertrophy, and nasal tumours. 

When history is suggestive of AR and clinical findings further support the diagnosis of AR, the next step is to classify and assess severity of disease. Allergic Rhinitis and its Impact on Asthma (ARIA) classifies rhinitis by duration and by severity according to the effect on quality of life [1]. Alternatively, AR can be classified as seasonal or perennial, based on the relevant allergen [18]. Typical perennial allergens include house dust mite, moulds, and animal dander. Both classification systems are useful in routine clinical practice, and provide supplementary information, especially when classifying rhinitis based on the type of allergen is not applicable. Moreover, ARIA offers treatment algorithms with step-up and step-down options [1]. Nevertheless, whenever history or physical examination reveals features not typically found in AR, nasal examination with endoscopy should be performed.

## 5. Non-Allergic Rhinitis 

Detailed information regarding the prevalence and burden of NAR in children is lacking. This category includes children with chronic rhinitis whose symptoms cannot be attributed to AR and infectious rhinitis. The diagnosis of NAR is again based on a detailed medical history [3]. Symptoms may be continuously present or induced by exposure to special triggers, such as systematically or intranasal taken medications, gastroesophageal reflux, and hormones. Temperature and humidity changes, physical exercise, tobacco smoke, and perfumes are known for inducing an overall hyper responsiveness of nasal mucosa [31]. In a significant portion of patients, no clear aetiology can be identified (idiopathic rhinitis). Idiopathic rhinitis is considered a diagnosis of exclusion. NAR prevalence remains steady across different age groups in paediatric population [10]. Besides history and anterior rhinoscopy, nasal endoscopy is essential in this type of rhinitis where features are unclear. Nasal endoscopy allows us to inspect the whole nasal cavity and exclude presence of nasal polyps or other pathologies like infection, significant anatomic deformities, and nasal tumours. 

## 6. Rhinosinusitis

Physicians should be able to differentiate AR from CRS. It has been recently shown that chronic rhinosinusitis is not causally related with allergic rhinitis in children [32]. In paediatric population, CRS is defined as an inflammation of sinonasal mucosa characterized by two or more symptoms; one should be obstruction and/or discoloured secretion accompanied by frontal pain, headache, and/or cough [11,33]. In CRS, secretions are often purulent or mucopurulent. Symptoms may be bilateral but typically have a unilateral propensity. Headache and facial pressure can be moderate to severe and should be evaluated in the context of other nasal symptoms. Other symptoms may be poor smell, cough, and halitosis. Headache in the absence of nasal symptoms should not be attributed to CRS [11,15]. In anterior rhinoscopy, typical signs of sinonasal inflammation would be mucopurulent/purulent or discoloured secretions, mucosal oedema, redness, and polyps. Nasal endoscopy should be performed for a detailed evaluation of the nasal cavity. Nasal polyps in the paediatric population, especially massive polyposis, should raise suspicion of other systematic diseases such as cystic fibrosis, and immunodeficiency and should be investigated accordingly [11]. Persistence of purulent secretions despite treatment from birth should be checked for primary ciliary dyskinesia [11,15]. 

## 7. Conclusions

Herein, we highlighted the role of a detailed medical history and clinical examination in the diagnosis of rhinitis in the paediatric population. A thorough medical history in combination with physical examination is extremely useful in increasing diagnostic accuracy and excluding other pathologies. Nasal endoscopy is a valuable tool which should be used every time symptoms are not typical for AR, or the existence of comorbidities cannot be ruled out. It is considered acceptable to make the diagnosis of AR and start treatment based on history and physical examination, so as not to delay treatment initiation. In cases where treatment fails, we need to take a step back to re-evaluate our data and reconsider our diagnosis. Utilization of a symptom-based and age-related approach as well as some further tests may be necessary to evaluate other possible diagnoses in children. 

## Figures and Tables

**Table 1:** Mostcommon causes of chronic rhinitis in the paediatric population.

⮚ Allergic rhinitis (AR) ⮚ Infectious rhinitis ⮚ Non-allergic rhinitis (NAR) ◦ Drug-induced ◦ Hormonal-induced ◦ Gustatory rhinitis ◦ Idiopathic rhinitis ⮚ Local allergic rhinitis (LAR) ⮚ Mixed rhinitis ⮚ Structural abnormalities ⮚ Adenoidal hypertrophy ⮚ Immunodeficiency ⮚ Cystic fibrosis (CF) ⮚ Ciliary dyskinesia

**Table 2:** Basic questions framework for interviewing patients with rhinitis. HDM: house dust mite; CT: computed tomography; SPT: skin prick test; IgE: immunoglobulin E.

⮚ Age ⮚ Type, timing, duration, and frequency of symptoms experienced ⮚ Symptoms’ seasonal distribution (seasonal, perennial, perennial with seasonal exacerbations, sporadic) ⮚ Triggers eliciting nasal symptoms (pollen, HDM, grass, trees, moulds, pets, etc.) ⮚ Environmental exposures eliciting symptoms at home/school (tobacco, chemicals, etc.) ⮚ Medications or other measures that relieve or exacerbate symptoms ⮚ Comorbidities (conjunctivitis, asthma, obstructive sleep apnoea, snoring, oral breathing, pollen-food syndrome, hearing loss, etc.) ⮚ History of frequent ear, nasal, sinonasal, and systemic infections ⮚ History of nose picking ⮚ Impact of rhinitis on daily activities, school performance, leisure, sleep ⮚ Family history of atopy ⮚ Prior allergy testing history (SPT, specific serum IgE) ⮚ Prior CT imaging or other diagnostic work up ⮚ Prior surgical history (ear tubes, adenoidectomy, tonsillectomy, sinus surgery, turbinoplasty) ⮚ Atypical symptoms. *Check for unilateral symptoms, pain, recurrent epistaxis, nasal obstruction without other symptoms, mucopurulent rhinorrhea, thick post nasal drip, and recurrent, prolonged infections*

**Table 3:** Differentiating symptoms and signs of rhinitis in children.

Clinical Findings	Underlying Pathology
Watery rhinorrhea	Common cold, AR, NAR, CSF leakage (more often unilateral especially with a history of trauma or surgery in the head or skull base)
Discoloured secretion purulent/mucopurulent discharge	Infectious rhinitis, CRS, NAR, adenoidal hypertrophy, foreign body (often unilateral), choanal atresia, immunodeficiency, PCD
Sneezing	AR (especially paroxysmal)
Polyps unilateral	Encephalocele, antrochoanal polyp, CRS, other benign and malignant tumours
Polyps bilateral	CRS, CF
Mouth breathing	AR, CRS, adenotonsillar disease, adenoidal hypertrophy, CF, choanal atresia, benign and malignant tumours
Allergic crease, shiner, Dennie–Morgan lines	AR, adenoidal hypertrophy
Epistaxis	Benign and malignant nasal tumours, coagulopathy
Retracted TM, COM with effusion	Adenoid hypertrophy, AR

Abbreviations: CSF leakage: cerebrospinal fluid leakage; CRS: chronic rhinosinusitis; PCD: Primary Ciliary Dyskinesia; CF: Cystic Fibrosis; TM: Tympanic membrane; COM: Chronic Otitis Media.
